# Maternal Genes and Facial Clefts in Offspring: A Comprehensive Search for Genetic Associations in Two Population-Based Cleft Studies from Scandinavia

**DOI:** 10.1371/journal.pone.0011493

**Published:** 2010-07-09

**Authors:** Astanand Jugessur, Min Shi, Håkon Kristian Gjessing, Rolv Terje Lie, Allen James Wilcox, Clarice Ring Weinberg, Kaare Christensen, Abee Lowman Boyles, Sandra Daack-Hirsch, Truc Trung Nguyen, Lene Christiansen, Andrew Carl Lidral, Jeffrey Clark Murray

**Affiliations:** 1 Division of Epidemiology, Norwegian Institute of Public Health, Oslo, Norway; 2 Craniofacial Research, Musculoskeletal Disorders, Murdoch Childrens Research Institute, Royal Children's Hospital, Parkville, Australia; 3 Biostatistics Branch, National Institute of Environmental Health Sciences (NIEHS), Research Triangle Park, Durham, North Carolina, United States of America; 4 Department of Public Health and Primary Health Care, University of Bergen, Bergen, Norway; 5 Medical Birth Registry of Norway, Norwegian Institute of Public Health, Bergen, Norway; 6 Epidemiology Branch, National Institute of Environmental Health Sciences (NIEHS), Research Triangle Park, North Carolina, United States of America; 7 Department of Epidemiology, University of Southern Denmark, Odense, Denmark; 8 College of Nursing, University of Iowa, Iowa City, Iowa, United States of America; 9 Departments of Pediatrics, Epidemiology and Biological Sciences, University of Iowa, Iowa City, Iowa, United States of America; Leiden University Medical Center, Netherlands

## Abstract

**Background:**

Fetal conditions can in principle be affected by the mother's genotype working through the prenatal environment.

**Methodology/Principal Findings:**

Genotypes for 1536 SNPs in 357 cleft candidate genes were available from a previous analysis in which we focused on fetal gene effects [1]. After data-cleaning, genotypes for 1315 SNPs in 334 autosomal genes were available for the current analysis of maternal gene effects. Two complementary statistical methods, TRIMM and HAPLIN, were used to detect multi-marker effects in population-based samples from Norway (562 case-parent and 592 control-parent triads) and Denmark (235 case-parent triads). We analyzed isolated cleft lip with or without cleft palate (iCL/P) and isolated cleft palate only (iCP) separately and assessed replication by looking for genes detected in both populations by both methods. In iCL/P, neither TRIMM nor HAPLIN detected more genes than expected by chance alone; furthermore, the selected genes were not replicated across the two methods. In iCP, however, *FLNB* was identified by both methods in both populations. Although *HIC1* and *ZNF189* did not fully satisfy our stringency criterion for replication, they were strongly associated with iCP in TRIMM analyses of the Norwegian triads.

**Conclusion/Significance:**

Except for *FLNB*, *HIC1* and *ZNF189*, maternal genes did not appear to influence the risk of clefting in our data. This is consistent with recent epidemiological findings showing no apparent difference between mother-to-offspring and father-to-offspring recurrence of clefts in these two populations. It is likely that fetal genes make the major genetic contribution to clefting risk in these populations, but we cannot rule out the possibility that maternal genes can affect risk through interactions with specific teratogens or fetal genes.

## Introduction

With an average worldwide prevalence of 1/800 live births, orofacial clefts are among the most common human birth defects [2]. Even with corrective surgery, patients face a lifetime of functional, social, and aesthetic challenges. Clefting has also been linked to an elevated risk of cancer in later life [3], and an increased overall mortality well into adulthood [4]. Despite significant progress in the identification of genetic and environmental risk factors for clefting [5], [6], the vast majority of isolated cleft cases still have no known cause.

Fetal conditions may be caused by the fetal genotype, and also by the mother's genotype working through the prenatal environment. Under assumed genetic mating symmetry, contributions through the maternal genotype would be apparent as over-representation of risk alleles in the maternal compared to the paternal genotype, among parents of affected offspring. Such maternally-mediated effects could confound a case-control study of fetal effects, due to correlation between the baby's and mother's genotype. To help disentangle offspring-mediated gene effects from those that are maternally-mediated, powerful analytic tools have been devised based on offspring-parent triads. Such methods test for the asymmetric distribution of a particular risk allele/haplotype between mothers and fathers (to detect maternally-mediated effects), and between affected offspring and their biologic parents (to detect offspring-mediated effects) [7], [8], [9]. We used two such methods, TRIMM [10] and HAPLIN [7], applied to two large population-based samples from Scandinavia (Norway and Denmark), and using one of the largest available collections of cleft candidate genes, in order to investigate whether maternal genes influence the fetal risk of iCL/P and iCP.

## Materials and Methods

### Participants

A population-based case-control study of orofacial clefts in Norway (1996–2001) provided 562 case-parent triads and 592 control-parent triads for analysis. Of the 562 case-parent triads, 311 were iCL/P and 114 were iCP. An additional 183 iCL/P and 69 iCP case-parent triads were available from a population-based study of orofacial clefts in Denmark (1991–2001). Details regarding study design and characteristics of study participants have been provided elsewhere [11], [12].

### Data analysis

Genotypes for 1536 SNPs in 357 cleft candidate genes were available from a previous analysis in which we searched for fetal gene effects in the same dataset [1]. After data-cleaning and exclusion of SNPs on the X-chromosome, genotypes for 1315 SNPs in 334 autosomal genes were available for the current analysis of maternal gene effects. We categorized these genes according to functional group and biological pathway to enable a biologically more meaningful interpretation of the results (for a complete list of these genes and pathways, see **Table S1**). Two statistical software packages, TRIMM [10] and HAPLIN [7], were used to scan for associations in the Norwegian and Danish samples. Both methods were designed to detect fetal or maternal gene effects separately using case-parent triads, although in different ways [1]. To assess a potential maternally-mediated gene effect, TRIMM constructs a difference vector by taking the genotype differences between the mother and the father. Under the genetic mating symmetry assumption [13], the difference vector has an expected value of 0 at each locus under the null hypothesis. TRIMM is nonparametric and generates a combined p-value by integrating results from two complementary tests: max-Z and Hotelling's T^2^. HAPLIN is a haplotype-based extension of the log-linear modeling approach [9] and uses maximum likelihood to estimate and test for maternal gene effects under the same genetic mating symmetry assumption. The missing phase information is accommodated by use of the Expectation-Maximization algorithm [7]. It is worth noting that effects of maternal genes are not confounded by effects of fetal genes in either of the methods.

TRIMM is nonparametric and can accommodate population structure, deviation from Hardy-Weinberg equilibrium, multiple SNPs, missing SNPs, and non-negligible recombination rates. When applied to a set of SNPs within a gene, TRIMM accounts for within-gene SNP correlations by permuting alleles at all SNPs simultaneously. In contrast, HAPLIN is parametric and estimates the full haplotype distribution over a set of SNPs and also provides estimates of relative risk for each haplotype. HAPLIN produces a complete description of the ‘risk structure’ over the set of haplotypes in a region through the use of a full maximum likelihood model. Compared with TRIMM, HAPLIN requires Hardy-Weinberg equilibrium and assumes no recombination. In the current analyses, we used three SNPs in a sliding-window haplotype analysis of the iCL/P and iCP case-parent triads. Longer window-lengths may generate many rare (and perhaps irrelevant) haplotypes, particularly when the sample size is limited. Details on the sliding-window approach and adjustment of the resulting p-values for within-gene multiple testing have been provided in [1].

HAPLIN handles incomplete triads by implementing a maximum likelihood model and by using the expectation maximization (EM) algorithm to impute missing triads. The estimated p-values and confidence intervals are subsequently adjusted to account for the imputation procedure itself. To ensure that maternal gene effects will not be confounded with fetal gene effects, TRIMM uses only complete triads. For randomly missing genotypes, TRIMM replaces the corresponding difference vector with zero—the expected value under the null.

We opted not to combine the Norwegian and Danish samples (although this would have boosted statistical power) because we had no prior reason to believe that the same genetic variants or haplotypes contribute to the risk of isolated clefts in both populations. In fact, our recent analysis of fetal gene effects in the same two study populations showed evidence of across-population differences [1]. Thus, TRIMM and HAPLIN analyses were performed separately on the Norwegian and Danish iCL/P and iCP triads (a total of four sets of analyses). We assessed the distribution of the resulting p-values from these analyses using a Schweder-Spjøtvoll plot [14], which is a simple graphical procedure for the simultaneous evaluations of many tests. In the absence of association, the observed p-values are expected to fall along the sloping line representing the uniform distribution under the null. For genes that are truly associated with disease, the corresponding p-values are expected to deviate from this sloping line.

As an alternative to correcting for multiple-testing using a full Bonferroni correction, we used quantile-quantile (QQ) plots to visually inspect whether our analyses produced more significant results than would have been expected by chance alone. QQ plots were generated for each cleft type (iCL/P and iCP) after p-values from the Norwegian and Danish analyses were combined using Fisher's method [15]. If the distribution of p-values is identical to the null distribution (for no association), points in the QQ plot are expected to follow the uniform diagonal line. Conversely, large-effect susceptibility loci are expected to deviate from the uniform distribution at the highly significant end of the distribution.

Finally, to verify whether the genetic mating symmetry assumption [13] holds in our data, we compared the QQ plots for the maternal gene effect analyses on the Norwegian control-parent triads with those on the case-parent triads. The rationale is that if the control results show departures from uniformity that are as inflated as those for the case-parent triad analyses, then there may be some violations of mating symmetry in the population.

### Software

TRIMM and HAPLIN are available for the ***R*** computing environment [16] from our web sites (TRIMM: http://www.niehs.nih.gov/research/atniehs/labs/bb/staff/weinberg/index.cfm#downloads; HAPLIN: http://www.uib.no/smis/gjessing/genetics/software/haplin).

### Ethics approval

The Norwegian Data Inspectorate, the Regional Committee on Research Ethics for Western Norway, and the respective Institutional Review Boards of the US National Institute of Environmental Health Sciences (NIH/NIEHS) and the University of Iowa approved the study. Ethics approval for the Danish orofacial clefts study was obtained from the Danish National Committee on Biomedical Research Ethics. Clinicopathological information and biologic specimens for DNA extraction were obtained from all participating families with the informed consent of the mothers and fathers, and all aspects of this research were in compliance with the tenets of the *Declaration of Helsinki* for human research (http://www.wma.net).

## Results

**Figure 1:**
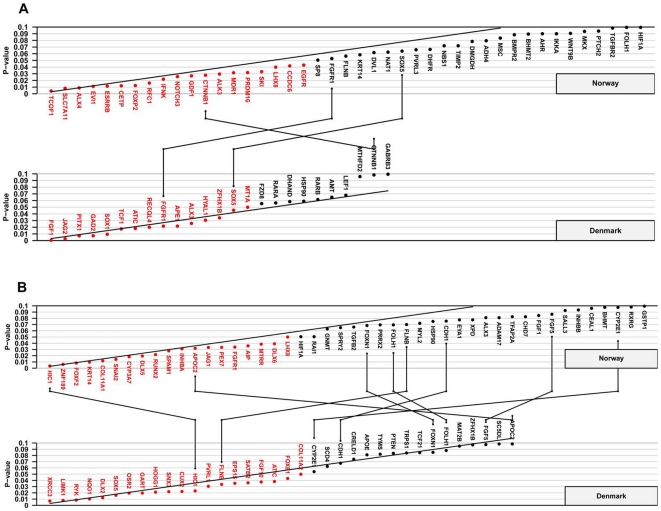
TRIMM analyses of the Norwegian and Danish samples. Schweder-Spjøtvoll plot of p-values for (**A**) isolated cleft lip with or without cleft palate (iCL/P) and (**B**) isolated cleft palate (iCP). All genes with p-values ≤0.1 are shown on the X-axis and ordered according to observed p-values (Y-axis). Genes with p-values ≤0.05 are highlighted in red. The sloping line represents the expected uniform distribution under the null (of no association). Genes with p-values ≤0.1 in both the Norwegian and Danish samples are indicated by lines connecting the upper (Norway) and lower (Denmark) plots.

**Figure 2:**
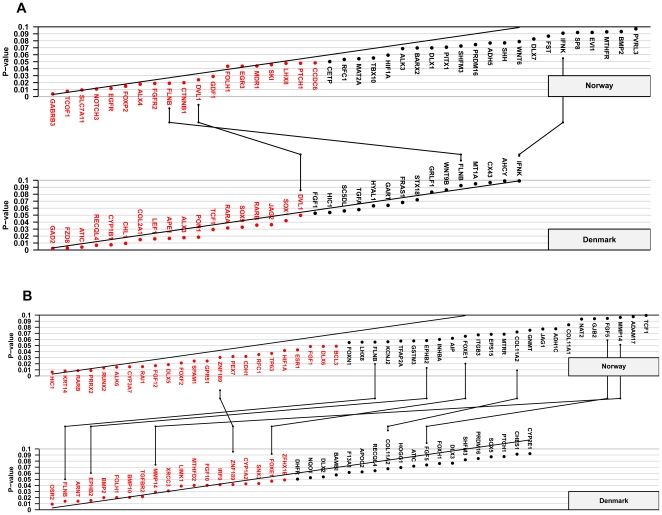
HAPLIN analyses of the Norwegian and Danish samples. Schweder-Spjøtvoll plot of p-values for (**A**) isolated cleft lip with or without cleft palate (iCL/P) and (**B**) isolated cleft palate (iCP). All genes with p-values ≤0.1 are shown on the X-axis and ordered according to observed p-values (Y-axis). Genes with p-values ≤0.05 are highlighted in red. The sloping line represents the expected uniform distribution under the null. Genes with p-values ≤0.1 in both the Norwegian and Danish samples are indicated by lines connecting the upper (Norway) and lower (Denmark) plots.

**Figures 1** and **2** represent Schweder-Spjøtvoll plots for all genes with p-values ≤0.1 from TRIMM and HAPLIN analyses of the Norwegian and Danish iCL/P and iCP samples, respectively. More detailed summaries of these results by analytic method, cleft type, and population are presented in **Tables S2, S3, S4, and S5**, as are Fisher-combined p-values for iCL/P and iCP after separate TRIMM and HAPLIN analyses in each population.

To evaluate replication, we looked at genes that achieved a p-value ≤0.1 in both Norway and Denmark. If the 334 genes were all unlinked, one would expect about 3 genes (0.1×0.1×334 genes) to ‘replicate’ by chance alone. For TRIMM and HAPLIN analyses of iCL/P, there were exactly 3 genes that replicated in this manner (**Figures 1A** and **2A**; pairs of identical genes are linked by lines joining the two plots). For iCP, there were 8 genes shared in the two samples in the TRIMM analysis, and 7 shared genes in the HAPLIN analysis (**Figures 1B** and **2B**). While this is more than the 3 expected by chance, there was only one gene, *FLNB* (filamin B, beta), that was replicated by both methods across both populations.

The genes for hypermethylated in cancer 1 (*HIC1*) and zinc finger protein 189 (*ZNF189*) did not fully meet our stringency criterion for replication (both methods detecting associations in both populations). Nevertheless, they were the top two genes associated with iCP in TRIMM analyses of the Norwegian triads (upper panel, **Figure 1B**). Only TRIMM found an association with *HIC1* in both populations (**Figure 1B**), while only HAPLIN detected an association with *ZNF189* in both populations (**Figure 2B**).

**Figure 3:**
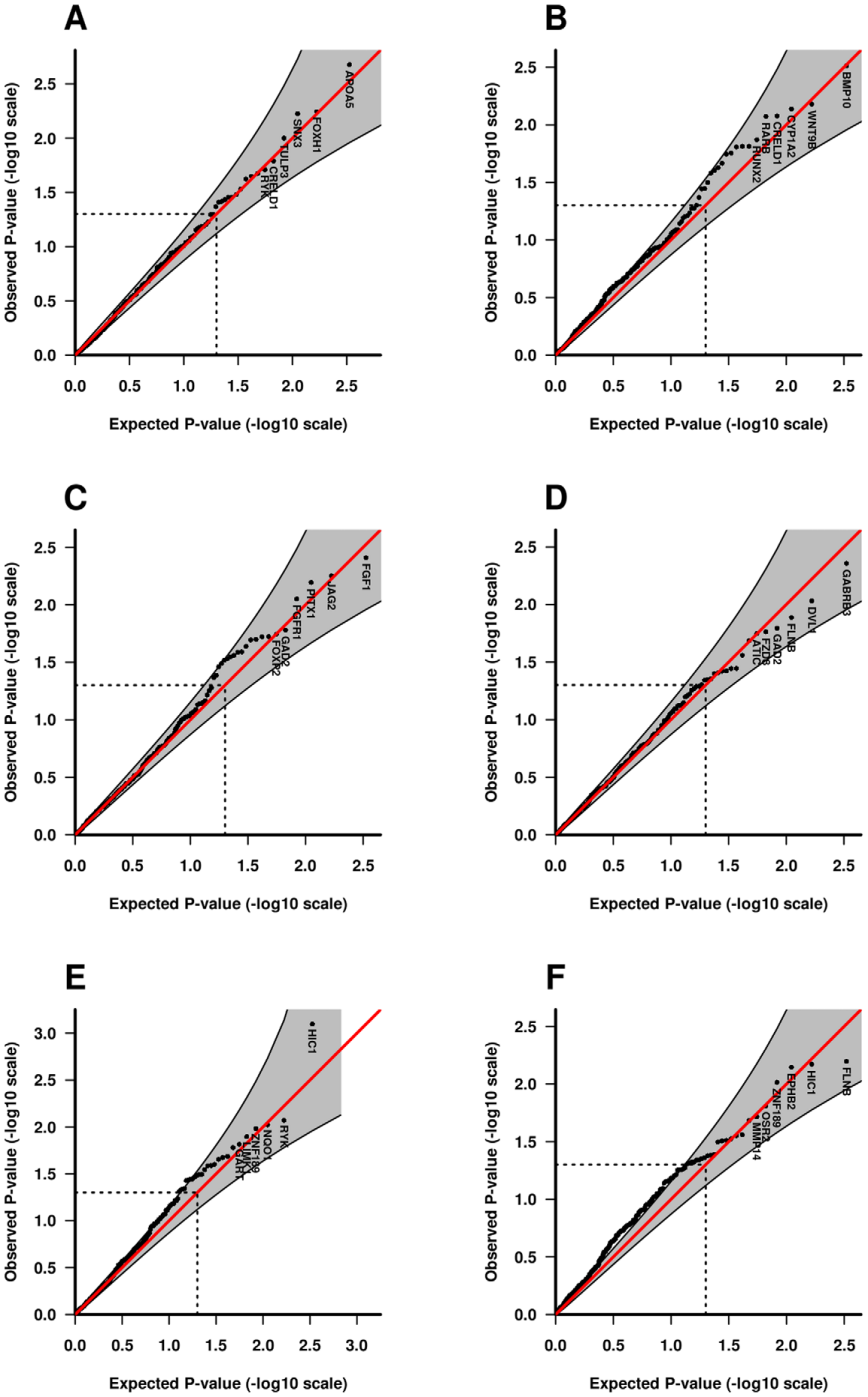
Quantile-quantile (QQ) plots of p-values. The QQ plots compare the distribution of the observed Fisher-combined p-values (-log_10_ scale) for both populations with an expected uniform distribution under the null (sloping line). The plots for the Norwegian control-parent triads, iCL/P triads, and iCP triads are provided separately for TRIMM in **A**, **C** and **E** respectively (left-hand side of the plot). The corresponding plots for HAPLIN are provided in **B**, **D** and **F** respectively (right-hand side of the plot). Gene labels for the top six most significant genes are displayed in each plot. Shaded areas represent 95% confidence interval bands and dotted lines indicate the expected ranked p-value of 0.05.

When the distribution of the observed Fisher-combined p-values was contrasted with that of the null, *HIC1* and *FLNB* in iCP analyses showed marked deviations at the significant end of the distributions in the corresponding QQ plots (**Figure 3**). Finally, to assess plausibility of the genetic mating symmetry assumption, we compared QQ plots for case-parent triads with those for the Norwegian control-parent triads. The QQ plots for the Norwegian control-parent triads (**Figure 3**) do not look different from a typical null plot and thus support the genetic mating symmetry assumption.

## Discussion

Our search for maternally-mediated genetic risk of orofacial clefts in offspring was motivated by evidence from animal studies demonstrating an ability of the products of maternal genes to directly intervene and protect the fetus. For example, Letterio et al. [17] showed that maternal Tgfb1 was able to cross the placenta and rescue *Tgfb1* ^−/−^ mice. Similar observations were made in an earlier experiment that tested whether maternal epidermal growth factor (Egf) could be transported to the fetus via the placenta [18]. In humans, however, evidence for maternal gene effects on the risk of clefting is less direct, and the few published studies are primarily single-gene association analyses that provide only a partial assessment of risk. To address this limitation, we focused here on broader gene categories and pathways, including the cholesterol pathway, the folate/homocysteine pathway, and genes involved in the detoxification of xenobiotic compounds.

Of the large number of candidate genes tested in this study, only *FLNB* was detected by both methods and across both populations in the iCP cleft category. This gene belongs to a family of actin-binding proteins that are highly conserved and widely expressed during development [19]. Filamins were discovered as the first family of non-muscle actin-binding proteins [20]. They stabilize the cytoskeletal network by cross-linking actin, and thus linking the cell membrane to the cytoskeleton, and by providing scaffolds on which intracellular signaling and protein trafficking pathways are organized [21], [22]. In humans, mutations in *FLNB* disrupt vertebral segmentation, joint formation, and skeletogenesis [21]. Examples of skeletal disorders include boomerang dysplasia, autosomal-recessive spondylocarpotarsal syndrome, autosomal-dominant Larsen syndrome, and the perinatal lethal atelosteogenesis phenotypes I and III [22], [23]. Interestingly, many of the filaminopathies manifest *cleft palate* as part of the overall phenotype [24], [25], which is consistent with our findings of an association of *FLNB* in iCP alone and not in the larger sample of iCL/P.

Unlike *FLNB*, *HIC1* and *ZNF189* did not fully meet our stringency criterion for replication, despite being the top two genes associated with iCP in TRIMM analyses of the Norwegian triads. This may be due to the small size of the Danish iCP triads (69 iCP case-parent triads), which may have limited the power to detect an association. Nonetheless, both of these genes are plausible candidates for orofacial clefting. *HIC1* encodes a zinc-finger transcription factor and maps to chromosome 17p13.3, within a 350 kb region found to be deleted in most patients with Miller-Dieker lissencephaly syndrome (MDLS) [26], [27], [28], [29]. Patients with MDLS exhibit a range of developmental anomalies, including omphalocele, limb and digit defects, and craniofacial dismorphology. Further, mice lacking *Hic1* die perinatally and have cleft palate among a range of developmental defects [30], [31]. The fact that *Hic1* ^−/−^ mice exhibit cleft palate is noteworthy, given that the association of *HIC1* was confined to iCP in our data (with no association seen in the larger iCL/P sample). *HIC1* is also a potential tumor suppressor gene; it is frequently hypermethylated and its expression is downregulated in several types of cancer [29]. The link to cancer is noteworthy given the higher risk of cancer reported among parents whose first liveborn child had cleft lip/palate [3].

*ZNF189* maps to chromosome 9q22–q31 and encodes a Krüppel-like zinc finger protein. Recent genome-wide linkage analyses of a large number of multiplex families from diverse populations uncovered a highly significant linkage signal to the 9q22–q33 region encompassing *ZNF189* [32], [33], [34]. Although several important candidate genes for clefts have been characterized in this region (e.g. human homolog of *patched* (*PTCH* [35]), receptor tyrosine kinase-like orphan receptor 2 (*ROR2* [36], [37]), transforming growth factor beta receptor type 1 (*TGFBR1* [38]), and forkhead box E1 (*FOXE1* [33], [39], [40], [41])), *ZNF189* has not previously been linked with clefting.

Our study was based on two national cleft cohorts of similar ancestry, two complementary and robust statistical methods, and a large panel of SNPs in one of the largest available collections of cleft candidate genes. Despite the breadth of our approach, there was little evidence to suggest that maternal genes influenced the risk of iCL/P or iCP in our data. This apparent lack of maternal gene effects is consistent with recent epidemiological data on familial patterns of recurrence of orofacial clefts. If maternal genes had an impact on the risk of clefting through effects on the uterine environment, mother-to-offspring recurrence would be higher than father-to-offspring recurrence. However, mother-to-offspring recurrence of clefts was not statistically different from father-to-offspring recurrence in either Norway or Denmark [42], [43]. There was no statistically significant difference either between parent-to-offspring and sibling-to-sibling recurrence, suggesting that fetal genes alone are more likely to explain the majority of genetic risk for orofacial clefts.

Our results are also consistent with those of a larger study that investigated whether half sibships ascertained through an affected proband had a higher risk of clefts when the mother was the common parent [44]. A higher occurrence of clefting would be expected if a major maternal effect exists, but no such evidence was found in that study. Finally, in our recent pathway-wide analysis of maternal genes and the risk of CL/P and CP among 29 genes involved in folate/one-carbon metabolism, we found no convincing indication that genetic variants in these folate metabolism genes play an etiologic role in orofacial clefting [45].

It is also possible that maternal genes alone do not confer risk of clefts to the newborn unless specific environmental exposures are also present. For example, a reduced capacity of mothers to biotransform toxins due to a genetic susceptibility has been proposed as a plausible explanation for the adverse effects of smoking and alcohol consumption on pregnancy outcomes [46], [47]. A non-additive interaction may be triggered only when the mothers are exposed to smoking or alcohol during the first trimester of pregnancy. In addition, interactions between the maternal and fetal genotype cannot be ruled out. Such interactions should be evident as ‘main effects’ of the participating genotypes, but the magnitude of the apparent effect would be blunted without accounting for the etiologic cofactor, making these effects difficult to detect.

In conclusion, with the possible exception of *FLNB*, *HIC1* and *ZNF189*, our data suggest that maternal genes do not contribute significantly to orofacial clefting in the Norwegian and Danish samples. This is consistent with recent reports on familial patterns of recurrence of orofacial clefts. It is likely that fetal genes explain the majority of genetic risk for orofacial clefts in these two populations. However, our study does not rule out the possibility that maternal genes may affect risk through interactions with specific teratogens or fetal genes.

## Supporting Information

Table S1 334 autosomal cleft candidate genes broadly categorized into functional groups and biological pathways.
(0.36 MB DOC)

Table S2 TRIMM results for iCL/P.
(0.08 MB DOC)

Table S3 TRIMM results for iCP.
(0.09 MB DOC)

Table S4 HAPLIN results for iCL/P.
(0.08 MB DOC)

Table S5 HAPLIN results for iCP.
(0.10 MB DOC)
