# Supplementary material for: Maternal Genes and Facial Clefts in Offspring: A Comprehensive Search for Genetic Associations in Two Population-Based Cleft Studies from Scandinavia
Source: PLoS One. 2010 Jul 9;5(7):e11493. doi: 10.1371/journal.pone.0011493 (PMC2901336; doi:10.1371/journal.pone.0011493)
Supplement: Table S1 — 334 autosomal cleft candidate genes broadly categorized into functional groups and biological pathways. (0.36 MB DOC) [file pone.0011493.s001.doc]

**Table S1.** 334 autosomal cleft candidate genes broadly categorized into functional groups and biological pathways.

| Gene ID_Chromosome | Number of SNPs/gene | Gene category ^a^ |
| --- | --- | --- |
| *ABCA1_9* | 8 | CHOLESTEROL PATHWAY |
| *ACTN1_14* | 2 | CELL ADHESION |
| *ADAM17_2* | 7 | MATRIX METALLOPROTEINASES |
| *ADH1A_4* | 3 | DETOXIFICATION-RELATED GENES |
| *ADH1B_4* | 4 | DETOXIFICATION-RELATED GENES |
| *ADH1C_4* | 1 | DETOXIFICATION-RELATED GENES |
| *ADH4_4* | 5 | DETOXIFICATION-RELATED GENES |
| *ADH5_4* | 4 | DETOXIFICATION-RELATED GENES |
| *AHCY_20* | 2 | FOLATE/HOMOCYSTEINE PATHWAY RELATED GENES |
| *AHR_7* | 4 | DETOXIFICATION-RELATED GENES |
| *AIP_11* | 3 | DETOXIFICATION-RELATED GENES |
| *ALDH1A1_9* | 8 | DETOXIFICATION-RELATED GENES |
| *ALK3_10* | 6 | OTHER CELL SIGNALLING MOLECULES |
| *ALK6_4* | 6 | OTHER CELL SIGNALLING MOLECULES |
| *ALX3_1* | 5 | TRANSCRIPTION FACTORS, REPRESSORS AND ACTIVATORS |
| *ALX4_11* | 1 | TRANSCRIPTION FACTORS, REPRESSORS AND ACTIVATORS |
| *AMT_3* | 3 | NA |
| *APE1_14* | 4 | DNA REPAIR |
| *APOA1_11* | 1 | CHOLESTEROL PATHWAY |
| *APOA1BP_1* | 1 | CHOLESTEROL PATHWAY |
| *APOA5_11* | 2 | CHOLESTEROL PATHWAY |
| *APOB_2 1* | 11 | CHOLESTEROL PATHWAY |
| *APOC2_19* | 3 | CHOLESTEROL PATHWAY |
| *APOE_19* | 3 | CHOLESTEROL PATHWAY |
| *ARNT_1* | 4 | DETOXIFICATION-RELATED GENES |
| *ARNT2_15* | 4 | DETOXIFICATION-RELATED GENES |
| *ARVCF_22* | 4 | GENES FOR SYNDROMES INCLUDING CLEFTS |
| *ATIC_2* | 4 | NA |
| *ATR_3* | 6 | DNA REPAIR |
| *BAMBI_10* | 4 | OTHER CELL SIGNALLING MOLECULES |
| *BARX1_9* | 2 | TRANSCRIPTION FACTORS, REPRESSORS AND ACTIVATORS |
| *BARX2_11* | 5 | TRANSCRIPTION FACTORS, REPRESSORS AND ACTIVATORS |
| *BCL3_19* | 2 | NA |
| *BHMT_5* | 3 | FOLATE/HOMOCYSTEINE PATHWAY RELATED GENES |
| *BHMT2_5* | 2 | FOLATE/HOMOCYSTEINE PATHWAY RELATED GENES |
| *BMP10_2* | 3 | OTHER CELL SIGNALLING MOLECULES |
| *BMP2_20* | 3 | OTHER CELL SIGNALLING MOLECULES |
| *BMP4_14* | 2 | OTHER CELL SIGNALLING MOLECULES |
| *BMP6_6* | 7 | OTHER CELL SIGNALLING MOLECULES |
| *BMPR2_2* | 4 | OTHER CELL SIGNALLING MOLECULES |
| *CASR_3* | 6 | OTHER CELL SIGNALLING MOLECULES |
| *CBS_21* | 2 | FOLATE/HOMOCYSTEINE PATHWAY RELATED GENES |
| *CCR1_3* | 1 | OTHER CELL SIGNALLING MOLECULES |
| *CCR6_6* | 2 | OTHER CELL SIGNALLING MOLECULES |
| *CCT3_1* | 2 | OTHER CELL SIGNALLING MOLECULES |
| *CDH1_16* | 3 | CELL ADHESION |
| *CDH2_18* | 5 | CELL ADHESION |
| *CDKN1C_11* | 4 | OTHER CELL SIGNALLING MOLECULES |
| *CEAL1_19* | 2 | NA |
| *CETP_16* | 2 | CHOLESTEROL PATHWAY |
| *CFC1_2* | 1 | GROWTH FACTORS AND RECEPTORS |
| *CHD7_8* | 2 | GENES FOR SYNDROMES INCLUDING CLEFTS |
| *CHES1_14* | 8 | TRANSCRIPTION FACTORS, REPRESSORS AND ACTIVATORS |
| *CHL1_3* | 5 | TRANSCRIPTION FACTORS, REPRESSORS AND ACTIVATORS |
| *CHRNA4_20* | 3 | NEUROTRANSMITTERS AND RELATED GENES |
| *CKM_19* | 3 | NA |
| *CLPTM1_19* | 3 | NA |
| *COL11A1_1* | 7 | CELL ADHESION |
| *COL11A2_6* | 6 | CELL ADHESION |
| *COL2A1_12* | 5 | CELL ADHESION |
| *CORS26_5* | 2 | NA |
| *CRABP1_15* | 2 | OTHER CELL SIGNALLING MOLECULES |
| *CRELD1_3* | 2 | GROWTH FACTORS AND RECEPTORS |
| *CTH_1* | 4 | FOLATE/HOMOCYSTEINE PATHWAY RELATED GENES |
| *CTNNB1_3* | 4 | OTHER CELL SIGNALLING MOLECULES |
| *CUX2_12* | 4 | TRANSCRIPTION FACTORS, REPRESSORS AND ACTIVATORS |
| *CX43_6* | 2 | NEUROTRANSMITTERS AND RELATED GENES |
| *CYP1A1_15* | 4 | DETOXIFICATION-RELATED GENES |
| *CYP1A2_15* | 2 | DETOXIFICATION-RELATED GENES |
| *CYP1B1_2* | 5 | DETOXIFICATION-RELATED GENES |
| *CYP2D6_22* | 3 | DETOXIFICATION-RELATED GENES |
| *CYP2E1_10* | 4 | DETOXIFICATION-RELATED GENES |
| *CYP3A7_7* | 2 | DETOXIFICATION-RELATED GENES |
| *DHAND_4* | 2 | TRANSCRIPTION FACTORS, REPRESSORS AND ACTIVATORS |
| *DHCR24_1* | 3 | CHOLESTEROL PATHWAY |
| *DHCR7_11* | 1 | CHOLESTEROL PATHWAY |
| *DHFR_5* | 2 | FOLATE/HOMOCYSTEINE PATHWAY RELATED GENES |
| *DKK1_10* | 3 | TRANSCRIPTION FACTORS, REPRESSORS AND ACTIVATORS |
| *DLX1_2* | 3 | TRANSCRIPTION FACTORS, REPRESSORS AND ACTIVATORS |
| *DLX2_2* | 2 | TRANSCRIPTION FACTORS, REPRESSORS AND ACTIVATORS |
| *DLX5_7* | 3 | TRANSCRIPTION FACTORS, REPRESSORS AND ACTIVATORS |
| *DLX6_7* | 3 | TRANSCRIPTION FACTORS, REPRESSORS AND ACTIVATORS |
| *DLX7_17* | 3 | TRANSCRIPTION FACTORS, REPRESSORS AND ACTIVATORS |
| *DMGDH _5* | 8 | FOLATE/HOMOCYSTEINE PATHWAY RELATED GENES |
| *DSP_6* | 5 | NA |
| *DTDST_5* | 5 | OTHER CELL SIGNALLING MOLECULES |
| *DVL1_1* | 2 | TRANSCRIPTION FACTORS, REPRESSORS AND ACTIVATORS |
| *EDN1_6* | 4 | CELL ADHESION |
| *EGF_4* | 5 | GROWTH FACTORS AND RECEPTORS |
| *EGFR_7* | 5 | GROWTH FACTORS AND RECEPTORS |
| *EGR3_8* | 3 | TRANSCRIPTION FACTORS, REPRESSORS AND ACTIVATORS |
| *EMX2_10* | 3 | TRANSCRIPTION FACTORS, REPRESSORS AND ACTIVATORS |
| *EPHB2_1* | 5 | CELL ADHESION |
| *EPHB3_3* | 4 | CELL ADHESION |
| *EPHX1_1* | 4 | DETOXIFICATION-RELATED GENES |
| *EPS15_1* | 4 | GROWTH FACTORS AND RECEPTORS |
| *ERCC4_16* | 3 | DNA REPAIR |
| *ESR1_6* | 8 | ESTROGEN RECEPTOR GENES |
| *ESR2_14* | 5 | ESTROGEN RECEPTOR GENES |
| *ESRRB_14* | 4 | ESTROGEN RECEPTOR GENES |
| *ETV5_3* | 5 | CELL ADHESION |
| *EVC_4* | 2 | GENES FOR SYNDROMES INCLUDING CLEFTS |
| *EVI1_3* | 4 | NA |
| *EYA1_8* | 7 | TRANSCRIPTION FACTORS, REPRESSORS AND ACTIVATORS |
| *F13A1_6* | 7 | NA |
| *FGF1_5* | 4 | GROWTH FACTORS AND RECEPTORS |
| *FGF10_5* | 4 | GROWTH FACTORS AND RECEPTORS |
| *FGF12_3* | 6 | GROWTH FACTORS AND RECEPTORS |
| *FGF2_4* | 7 | GROWTH FACTORS AND RECEPTORS |
| *FGF4_11* | 2 | GROWTH FACTORS AND RECEPTORS |
| *FGF5_4* | 2 | GROWTH FACTORS AND RECEPTORS |
| *FGF7_15* | 6 | GROWTH FACTORS AND RECEPTORS |
| *FGF8_10* | 5 | GROWTH FACTORS AND RECEPTORS |
| *FGF9_13* | 5 | GROWTH FACTORS AND RECEPTORS |
| *FGFBP1_4* | 2 | GROWTH FACTORS AND RECEPTORS |
| *FGFR1_8* | 6 | GROWTH FACTORS AND RECEPTORS |
| *FGFR2_10* | 4 | GROWTH FACTORS AND RECEPTORS |
| *FGFR3_4* | 2 | GROWTH FACTORS AND RECEPTORS |
| *FGFR4_5* | 4 | GROWTH FACTORS AND RECEPTORS |
| *FLNB_3* | 5 | CELL ADHESION |
| *FOLH1_11* | 4 | FOLATE/HOMOCYSTEINE PATHWAY RELATED GENES |
| *FOLR3_11* | 3 | FOLATE/HOMOCYSTEINE PATHWAY RELATED GENES |
| *FOLRA_11* | 2 | FOLATE/HOMOCYSTEINE PATHWAY RELATED GENES |
| *FOLRB_11* | 2 | FOLATE/HOMOCYSTEINE PATHWAY RELATED GENES |
| *FOXC2_16* | 3 | TRANSCRIPTION FACTORS, REPRESSORS AND ACTIVATORS |
| *FOXE1_9* | 6 | TRANSCRIPTION FACTORS, REPRESSORS AND ACTIVATORS |
| *FOXF2_6* | 3 | TRANSCRIPTION FACTORS, REPRESSORS AND ACTIVATORS |
| *FOXH1_8* | 3 | TRANSCRIPTION FACTORS, REPRESSORS AND ACTIVATORS |
| *FOXN1_17* | 2 | TRANSCRIPTION FACTORS, REPRESSORS AND ACTIVATORS |
| *FOXP2_7* | 5 | TRANSCRIPTION FACTORS, REPRESSORS AND ACTIVATORS |
| *FRAS1_4* | 7 | GENES FOR SYNDROMES INCLUDING CLEFTS |
| *FSCN1_7* | 1 | CELL ADHESION |
| *FST_5* | 3 | NA |
| *FTCD_21* | 3 | FOLATE/HOMOCYSTEINE PATHWAY RELATED GENES |
| *FTHFD_3* | 5 | FOLATE/HOMOCYSTEINE PATHWAY RELATED GENES |
| *FZD1_7* | 4 | NA |
| *FZD10_12* | 2 | NA |
| *FZD2_17* | 2 | TRANSCRIPTION FACTORS, REPRESSORS AND ACTIVATORS |
| *FZD4_11* | 3 | NA |
| *FZD7_2* | 4 | TRANSCRIPTION FACTORS, REPRESSORS AND ACTIVATORS |
| *FZD8_10* | 3 | TRANSCRIPTION FACTORS, REPRESSORS AND ACTIVATORS |
| *GABRB3_15* | 7 | NEUROTRANSMITTERS AND RELATED GENES |
| *GAD1_2* | 5 | NEUROTRANSMITTERS AND RELATED GENES |
| *GAD2_10* | 3 | NEUROTRANSMITTERS AND RELATED GENES |
| *GART_21* | 6 | NA |
| *GDF1_19* | 2 | OTHER CELL SIGNALLING MOLECULES |
| *GJB2_13* | 3 | NEUROTRANSMITTERS AND RELATED GENES |
| *GLI2_2* | 3 | TRANSCRIPTION FACTORS, REPRESSORS AND ACTIVATORS |
| *GLI3_7* | 6 | TRANSCRIPTION FACTORS, REPRESSORS AND ACTIVATORS |
| *GNMT _6* | 1 | FOLATE/HOMOCYSTEINE PATHWAY RELATED GENES |
| *GPR51_9* | 7 | NEUROTRANSMITTERS AND RELATED GENES |
| *GRLF1_19* | 3 | GLUCOCORTICOID RELATED |
| *GSTA4_6* | 4 | DETOXIFICATION-RELATED GENES |
| *GSTM1_1* | 2 | DETOXIFICATION-RELATED GENES |
| *GSTM3_1* | 3 | DETOXIFICATION-RELATED GENES |
| *GSTP1_11* | 3 | DETOXIFICATION-RELATED GENES |
| *GSTT1_22* | 1 | DETOXIFICATION-RELATED GENES |
| *HIC1_17* | 1 | OTHER CELL SIGNALLING MOLECULES |
| *HIF1A_14* | 5 | TRANSCRIPTION FACTORS, REPRESSORS AND ACTIVATORS |
| *HOGG1_3* | 2 | DNA REPAIR |
| *HOXA7_7* | 8 | TRANSCRIPTION FACTORS, REPRESSORS AND ACTIVATORS |
| *HOXB6_17* | 3 | TRANSCRIPTION FACTORS, REPRESSORS AND ACTIVATORS |
| *HSP90_14* | 1 | NA |
| *HYAL1_3* | 3 | NA |
| *IDH1_2* | 2 | CHOLESTEROL PATHWAY |
| *IFNK_9* | 3 | TRANSCRIPTION FACTORS, REPRESSORS AND ACTIVATORS |
| *IKKA_10* | 4 | TRANSCRIPTION FACTORS, REPRESSORS AND ACTIVATORS |
| *IKKE_1* | 3 | TRANSCRIPTION FACTORS, REPRESSORS AND ACTIVATORS |
| *INHBA_7* | 3 | OTHER CELL SIGNALLING MOLECULES |
| *INHBB_2* | 2 | OTHER CELL SIGNALLING MOLECULES |
| *IRF6_1* | 6 | TRANSCRIPTION FACTORS, REPRESSORS AND ACTIVATORS |
| *IRF9_14* | 2 | TRANSCRIPTION FACTORS, REPRESSORS AND ACTIVATORS |
| *ITGB3_17* | 2 | CELL ADHESION |
| *JAG1_20* | 4 | TRANSCRIPTION FACTORS, REPRESSORS AND ACTIVATORS |
| *JAG2_14* | 1 | TRANSCRIPTION FACTORS, REPRESSORS AND ACTIVATORS |
| *KCNJ2_17* | 2 | NA |
| *KREMEN1_22* | 4 | OTHER CELL SIGNALLING MOLECULES |
| *KRT14_17* | 3 | CELL ADHESION |
| *KRT18_12* | 2 | CELL ADHESION |
| *LCAT_16* | 1 | CHOLESTEROL PATHWAY |
| *LDLR_19* | 2 | CHOLESTEROL PATHWAY |
| *LEF1_4* | 5 | OTHER CELL SIGNALLING MOLECULES |
| *LEFTY2_1* | 7 | GROWTH FACTORS AND RECEPTORS |
| *LHX8_1* | 3 | TRANSCRIPTION FACTORS, REPRESSORS AND ACTIVATORS |
| *LIMK1_7* | 2 | TRANSCRIPTION FACTORS, REPRESSORS AND ACTIVATORS |
| *LIPC_15* | 5 | CHOLESTEROL PATHWAY |
| *LMX1B_9* | 2 | TRANSCRIPTION FACTORS, REPRESSORS AND ACTIVATORS |
| *LOR_1* | 3 | NA |
| *LPL_8* | 2 | CHOLESTEROL PATHWAY |
| *MARK4_19* | 2 | NEUROTRANSMITTERS AND RELATED GENES |
| *MAT1A_10* | 2 | OTHER CELL SIGNALLING MOLECULES |
| *MAT2A_2* | 4 | OTHER CELL SIGNALLING MOLECULES |
| *MAT2B_5* | 4 | OTHER CELL SIGNALLING MOLECULES |
| *MDCR_17* | 3 | GENES FOR SYNDROMES INCLUDING CLEFTS |
| *MDR1_7* | 5 | DETOXIFICATION-RELATED GENES |
| *MKX_10* | 4 | NA |
| *MMEL2_1* | 1 | NA |
| *MMP13_11* | 6 | MATRIX METALLOPROTEINASES |
| *MMP14_14* | 2 | MATRIX METALLOPROTEINASES |
| *MMP2_16* | 7 | MATRIX METALLOPROTEINASES |
| *MSC_8* | 4 | TRANSCRIPTION FACTORS, REPRESSORS AND ACTIVATORS |
| *MSX1_4* | 5 | TRANSCRIPTION FACTORS, REPRESSORS AND ACTIVATORS |
| *MSX2_5* | 4 | TRANSCRIPTION FACTORS, REPRESSORS AND ACTIVATORS |
| *MT1A_16* | 2 | MATRIX METALLOPROTEINASES |
| *MT4_16* | 3 | MATRIX METALLOPROTEINASES |
| *MTHFD1_14* | 3 | FOLATE/HOMOCYSTEINE PATHWAY RELATED GENES |
| *MTHFD2_2* | 1 | FOLATE/HOMOCYSTEINE PATHWAY RELATED GENES |
| *MTHFR_1* | 5 | FOLATE/HOMOCYSTEINE PATHWAY RELATED GENES |
| *MTHFS_15* | 3 | FOLATE/HOMOCYSTEINE PATHWAY RELATED GENES |
| *MTR_1* | 3 | FOLATE/HOMOCYSTEINE PATHWAY RELATED GENES |
| *MTRR_5* | 5 | FOLATE/HOMOCYSTEINE PATHWAY RELATED GENES |
| *MYL2_12* | 2 | NA |
| *NAT1_8* | 4 | DETOXIFICATION-RELATED GENES |
| *NAT2_8* | 7 | DETOXIFICATION-RELATED GENES |
| *NBS1_8* | 7 | GENES FOR SYNDROMES INCLUDING CLEFTS |
| *NIPBL_5* | 2 | TRANSCRIPTION FACTORS, REPRESSORS AND ACTIVATORS |
| *NNMT_11* | 7 | NA |
| *None_4* | 1 | NA |
| *NOTCH3_19* | 3 | TRANSCRIPTION FACTORS, REPRESSORS AND ACTIVATORS |
| *NQO1_16* | 3 | DETOXIFICATION-RELATED GENES |
| *NR3C1_5* | 6 | GLUCOCORTICOID RELATED |
| *NRXN2_11* | 2 | NA |
| *OSR2_8* | 1 | TRANSCRIPTION FACTORS, REPRESSORS AND ACTIVATORS |
| *PAX3_2* | 4 | TRANSCRIPTION FACTORS, REPRESSORS AND ACTIVATORS |
| *PAX8_2* | 2 | TRANSCRIPTION FACTORS, REPRESSORS AND ACTIVATORS |
| *PAX9_14* | 6 | TRANSCRIPTION FACTORS, REPRESSORS AND ACTIVATORS |
| *PDGFC_4* | 5 | GROWTH FACTORS AND RECEPTORS |
| *PDGFRA_4* | 4 | GROWTH FACTORS AND RECEPTORS |
| *PEX7_6* | 3 | NA |
| *PIPOX _17* | 2 | NA |
| *PITX1_5* | 2 | TRANSCRIPTION FACTORS, REPRESSORS AND ACTIVATORS |
| *PITX2_4* | 3 | TRANSCRIPTION FACTORS, REPRESSORS AND ACTIVATORS |
| *PKP1_1* | 4 | GENES FOR SYNDROMES INCLUDING CLEFTS |
| *POMT1_9* | 2 | NA |
| *PON1_7* | 8 | NA |
| *PRDM16_1* | 5 | GENES FOR SYNDROMES INCLUDING CLEFTS |
| *PRRX1_1* | 4 | TRANSCRIPTION FACTORS, REPRESSORS AND ACTIVATORS |
| *PRRX2_9* | 3 | TRANSCRIPTION FACTORS, REPRESSORS AND ACTIVATORS |
| *PTCH1_9* | 2 | NA |
| *PTCH2_1* | 2 | TRANSCRIPTION FACTORS, REPRESSORS AND ACTIVATORS |
| *PTEN_10* | 1 | OTHER CELL SIGNALLING MOLECULES |
| *PTPN11_12* | 4 | GENES FOR SYNDROMES INCLUDING CLEFTS |
| *PVR_19* | 1 | CELL ADHESION |
| *PVRL1_11* | 4 | CELL ADHESION |
| *PVRL2_19* | 5 | CELL ADHESION |
| *PVRL3_3* | 4 | CELL ADHESION |
| *RAI1_17* | 1 | OTHER CELL SIGNALLING MOLECULES |
| *RARA_17* | 2 | OTHER CELL SIGNALLING MOLECULES |
| *RARB_3* | 6 | OTHER CELL SIGNALLING MOLECULES |
| *RARG_12* | 3 | OTHER CELL SIGNALLING MOLECULES |
| *RET_10* | 2 | CELL ADHESION |
| *RFC1_4* | 3 | DNA REPAIR |
| *RIP3_14* | 2 | OTHER CELL SIGNALLING MOLECULES |
| *ROR1_1* | 5 | OTHER CELL SIGNALLING MOLECULES |
| *ROR2_9* | 6 | OTHER CELL SIGNALLING MOLECULES |
| *RUNX2_6* | 6 | TRANSCRIPTION FACTORS, REPRESSORS AND ACTIVATORS |
| *RXRG_1* | 5 | OTHER CELL SIGNALLING MOLECULES |
| *RYK_3* | 4 | GROWTH FACTORS AND RECEPTORS |
| *SALL1_16* | 3 | TRANSCRIPTION FACTORS, REPRESSORS AND ACTIVATORS |
| *SALL2_14* | 2 | TRANSCRIPTION FACTORS, REPRESSORS AND ACTIVATORS |
| *SALL3_18* | 4 | TRANSCRIPTION FACTORS, REPRESSORS AND ACTIVATORS |
| *SALL4_20* | 3 | TRANSCRIPTION FACTORS, REPRESSORS AND ACTIVATORS |
| *SARA1_10* | 3 | OTHER CELL SIGNALLING MOLECULES |
| *SATB2_2* | 7 | NA |
| *SC5DL_11* | 3 | TRANSCRIPTION FACTORS, REPRESSORS AND ACTIVATORS |
| *SCD4_4* | 6 | CHOLESTEROL PATHWAY |
| *SET_9* | 3 | OTHER CELL SIGNALLING MOLECULES |
| *SHFM3_10* | 5 | GENES FOR SYNDROMES INCLUDING CLEFTS |
| *SHH_7* | 3 | TRANSCRIPTION FACTORS, REPRESSORS AND ACTIVATORS |
| *SHMT1_17* | 1 | FOLATE/HOMOCYSTEINE PATHWAY RELATED GENES |
| *SIX3_2* | 1 | TRANSCRIPTION FACTORS, REPRESSORS AND ACTIVATORS |
| *SKI_1* | 1 | TRANSCRIPTION FACTORS, REPRESSORS AND ACTIVATORS |
| *SLC7A11_4* | 5 | OTHER CELL SIGNALLING MOLECULES |
| *SMAD1_4* | 4 | OTHER CELL SIGNALLING MOLECULES |
| *SMAD2_18* | 5 | OTHER CELL SIGNALLING MOLECULES |
| *SMAD3_15* | 6 | OTHER CELL SIGNALLING MOLECULES |
| *SMAD4_18* | 4 | OTHER CELL SIGNALLING MOLECULES |
| *SNAI1_20* | 5 | TRANSCRIPTION FACTORS, REPRESSORS AND ACTIVATORS |
| *SNAI2_8* | 6 | TRANSCRIPTION FACTORS, REPRESSORS AND ACTIVATORS |
| *SNX3_6* | 2 | OTHER CELL SIGNALLING MOLECULES |
| *SOX1_13* | 2 | TRANSCRIPTION FACTORS, REPRESSORS AND ACTIVATORS |
| *SOX5_12* | 8 | TRANSCRIPTION FACTORS, REPRESSORS AND ACTIVATORS |
| *SOX9_17* | 4 | TRANSCRIPTION FACTORS, REPRESSORS AND ACTIVATORS |
| *SP8_7* | 3 | TRANSCRIPTION FACTORS, REPRESSORS AND ACTIVATORS |
| *SPAM1_7* | 1 | CELL ADHESION |
| *SPP1_4* | 6 | GENES FOR SYNDROMES INCLUDING CLEFTS |
| *SPPL3_12* | 5 | OTHER CELL SIGNALLING MOLECULES |
| *SPRY2_13* | 2 | TRANSCRIPTION FACTORS, REPRESSORS AND ACTIVATORS |
| *SPTLC1_9* | 2 | GENES FOR SYNDROMES INCLUDING CLEFTS |
| *STAT3_17* | 2 | OTHER CELL SIGNALLING MOLECULES |
| *STX18_4* | 4 | NA |
| *SULT1A1_16* | 1 | DETOXIFICATION-RELATED GENES |
| *SUMO1_2* | 4 | TRANSCRIPTION FACTORS, REPRESSORS AND ACTIVATORS |
| *TBX1_22* | 6 | TRANSCRIPTION FACTORS, REPRESSORS AND ACTIVATORS |
| *TBX10_11* | 2 | TRANSCRIPTION FACTORS, REPRESSORS AND ACTIVATORS |
| *TBX15_1* | 1 | TRANSCRIPTION FACTORS, REPRESSORS AND ACTIVATORS |
| *TBX21_17* | 2 | TRANSCRIPTION FACTORS, REPRESSORS AND ACTIVATORS |
| *TBX4_17* | 3 | TRANSCRIPTION FACTORS, REPRESSORS AND ACTIVATORS |
| *TCF1_12* | 3 | TRANSCRIPTION FACTORS, REPRESSORS AND ACTIVATORS |
| *TCF21_6* | 2 | TRANSCRIPTION FACTORS, REPRESSORS AND ACTIVATORS |
| *TCOF1_5* | 2 | GENES FOR SYNDROMES INCLUDING CLEFTS |
| *TFAP2A_6* | 3 | TRANSCRIPTION FACTORS, REPRESSORS AND ACTIVATORS |
| *TGFA_2* | 4 | GROWTH FACTORS AND RECEPTORS |
| *TGFB1_19* | 3 | GROWTH FACTORS AND RECEPTORS |
| *TGFB2_1* | 6 | GROWTH FACTORS AND RECEPTORS |
| *TGFB3_14* | 3 | GROWTH FACTORS AND RECEPTORS |
| *TGFBR1_9* | 3 | GROWTH FACTORS AND RECEPTORS |
| *TGFBR2_3* | 5 | GROWTH FACTORS AND RECEPTORS |
| *TGFBR3_1* | 7 | GROWTH FACTORS AND RECEPTORS |
| *TGIF_18* | 4 | TRANSCRIPTION FACTORS, REPRESSORS AND ACTIVATORS |
| *THRB_3* | 7 | NA |
| *TIMP2_17* | 5 | MATRIX METALLOPROTEINASES |
| *TNFRSF10B_8* | 5 | OTHER CELL SIGNALLING MOLECULES |
| *TNNT3_11* | 3 | CELL ADHESION |
| *TP63_3* | 9 | GENES FOR SYNDROMES INCLUDING CLEFTS |
| *TRPS1_8 1* | 10 | GENES FOR SYNDROMES INCLUDING CLEFTS |
| *TULP3_12* | 3 | NA |
| *TWIST1_7* | 2 | TRANSCRIPTION FACTORS, REPRESSORS AND ACTIVATORS |
| *TYMS_18* | 4 | DNA REPAIR |
| *UFD1L_22* | 3 | GENES FOR SYNDROMES INCLUDING CLEFTS |
| *UGT1A7 _2* | 8 | DETOXIFICATION-RELATED GENES |
| *VCL_10* | 5 | CELL ADHESION |
| *WHSC1_4* | 1 | TRANSCRIPTION FACTORS, REPRESSORS AND ACTIVATORS |
| *WNT3A_1* | 2 | OTHER CELL SIGNALLING MOLECULES |
| *WNT4_1* | 6 | OTHER CELL SIGNALLING MOLECULES |
| *WNT5A_3* | 3 | OTHER CELL SIGNALLING MOLECULES |
| *WNT6 _2* | 2 | OTHER CELL SIGNALLING MOLECULES |
| *WNT7B_22* | 1 | OTHER CELL SIGNALLING MOLECULES |
| *WNT9B_17* | 3 | OTHER CELL SIGNALLING MOLECULES |
| *XPD_19* | 3 | DNA REPAIR |
| *XRCC1_19* | 5 | DNA REPAIR |
| *XRCC3_14* | 2 | DNA REPAIR |
| *ZFHX1B_2* | 5 | TRANSCRIPTION FACTORS, REPRESSORS AND ACTIVATORS |
| *ZNF189_9* | 5 | TRANSCRIPTION FACTORS, REPRESSORS AND ACTIVATORS |

^a^ NA, not available.
