# Supplementary material for: Maternal Genes and Facial Clefts in Offspring: A Comprehensive Search for Genetic Associations in Two Population-Based Cleft Studies from Scandinavia
Source: PLoS One. 2010 Jul 9;5(7):e11493. doi: 10.1371/journal.pone.0011493 (PMC2901336; doi:10.1371/journal.pone.0011493)
Supplement: Table S2 — TRIMM results for iCL/P. (0.08 MB DOC) [file pone.0011493.s002.doc]

**Table S2.** TRIMM results for iCL/P

| Gene ID ^a^ | Chromosome | Number of SNPs/gene | NORWAY iCL/P p-value ^b^ | DENMARK iCL/P p-value ^b^ | Fisher-combined p-value ^b, c^ |
| --- | --- | --- | --- | --- | --- |
| *FGF1* | 5 | 4 | 0.9485 | **0.0005** | **0.0039** |
| *JAG2* | 14 | 4 | 0.2485 | **0.0027** | **0.0056** |
| *PITX1* | 5 | 3 | 0.1080 | **0.0072** | **0.0064** |
| *FGFR1* | 8 | 6 | 0.0528 | **0.0217** | **0.0089** |
| *GAD2* | 10 | 3 | 0.3210 | **0.0073** | **0.0166** |
| *FOXP2* | 7 | 5 | **0.0122** | 0.2110 | **0.0180** |
| *CTNNB1* | 3 | 4 | **0.0279** | 0.0985 | **0.0189** |
| *EVI1* | 3 | 5 | **0.0110** | 0.2500 | **0.0190** |
| *SOX5* | 12 | 9 | 0.0640 | **0.0456** | **0.0199** |
| *IFNK* | 9 | 3 | **0.0219** | 0.1350 | **0.0202** |
| *TCOF1* | 5 | 3 | **0.0042** | 0.8185 | **0.0230** |
| *CETP* | 16 | 4 | **0.0119** | 0.3290 | **0.0257** |
| *ESRRB* | 14 | 4 | **0.0114** | 0.3465 | **0.0259** |
| *ATIC* | 2 | 4 | 0.2330 | **0.0182** | **0.0274** |
| *SLC7A11* | 4 | 5 | **0.0082** | 0.5395 | **0.0283** |
| *TCF1* | 12 | 4 | 0.2640 | **0.0175** | **0.0294** |
| *APE1* | 14 | 4 | 0.2210 | **0.0217** | **0.0304** |
| *ALX4* | 11 | 2 | **0.0088** | 0.5880 | **0.0325** |
| *SOX1* | 13 | 2 | 0.5890 | **0.0095** | **0.0346** |
| *ZFHX1B* | 2 | 5 | 0.2015 | **0.0341** | **0.0411** |
| *CCDC6* | 10 | 1 | **0.0418** | 0.1740 | **0.0430** |
| *ALX3* | 1 | 4 | 0.3600 | **0.0258** | 0.0527 |
| *HYAL1* | 3 | 2 | 0.4455 | **0.0306** | 0.0721 |
| *RFC1* | 4 | 3 | **0.0161** | 0.8520 | 0.0726 |
| *PRDM16* | 1 | 8 | **0.0318** | 0.5255 | 0.0851 |
| *RECQL4* | 8 | 3 | 0.9295 | **0.0200** | 0.0927 |
| *GDF1* | 19 | 3 | **0.0269** | 0.7060 | 0.0944 |
| *ALK3* | 10 | 7 | **0.0296** | 0.6765 | 0.0980 |
| *MDR1* | 7 | 3 | **0.0315** | 0.6510 | 0.1001 |
| *LHX8* | 1 | 4 | **0.0399** | 0.5615 | 0.1075 |
| *NOTCH3* | 19 | 3 | **0.0258** | 0.9230 | 0.1128 |
| *SKI* | 1 | 1 | **0.0331** | 0.9330 | 0.1382 |
| *EGFR* | 7 | 6 | **0.0430** | 0.9385 | 0.1700 |

^a^ Gene ID from NCBI Entrez Gene. Genes associated in both samples are boldfaced.

^b^ P-values ≤ 0.05 are boldfaced (the Fisher-combined p-values have not been Bonferroni-corrected).

^c^ The top six genes are shown in **Figure 3A**.
