# Supplementary material for: Maternal Genes and Facial Clefts in Offspring: A Comprehensive Search for Genetic Associations in Two Population-Based Cleft Studies from Scandinavia
Source: PLoS One. 2010 Jul 9;5(7):e11493. doi: 10.1371/journal.pone.0011493 (PMC2901336; doi:10.1371/journal.pone.0011493)
Supplement: Table S3 — TRIMM results for iCP. (0.09 MB DOC) [file pone.0011493.s003.doc]

**Table S3.** TRIMM results for iCP

| Gene ID ^a^ | Chromosome | Number of SNPs/gene | NORWAY iCP p-value ^b^ | DENMARK iCP p-value ^b^ | Fisher-combined p-value ^b, c^ |
| --- | --- | --- | --- | --- | --- |
| ***HIC1*** | 17 | 2 | **0.0035** | **0.0232** | **0.0008** |
| *RYK* | 3 | 4 | 0.1295 | **0.0084** | **0.0085** |
| *NQO1* | 16 | 3 | 0.1200 | **0.0102** | **0.0094** |
| *ZNF189* | 9 | 5 | **0.0061** | 0.2230 | **0.0104** |
| *LIMK1* | 7 | 3 | 0.2180 | **0.0079** | **0.0127** |
| *GART* | 21 | 6 | 0.1090 | **0.0197** | **0.0153** |
| *FLNB* | 3 | 5 | 0.0700 | **0.0336** | **0.0166** |
| *XRCC3* | 14 | 2 | 0.4400 | **0.0069** | **0.0206** |
| *APOC2* | 19 | 2 | **0.0312** | 0.0985 | **0.0212** |
| *CYP3A7* | 7 | 2 | **0.0183** | 0.1805 | **0.0222** |
| *EPS15* | 1 | 5 | 0.1075 | **0.0353** | **0.0250** |
| *CUX2* | 12 | 4 | 0.1770 | **0.0223** | **0.0258** |
| *SOX5* | 12 | 9 | 0.2455 | **0.0161** | **0.0259** |
| *SNAI2* | 8 | 6 | **0.0142** | 0.3140 | **0.0286** |
| *CDH1* | 16 | 4 | 0.0755 | 0.0675 | **0.0320** |
| *DLX5* | 7 | 4 | **0.0195** | 0.2630 | **0.0322** |
| *CYP2E1* | 10 | 5 | 0.0975 | 0.0540 | **0.0329** |
| *COL11A2* | 6 | 6 | 0.1110 | **0.0499** | **0.0343** |
| *FOXN1* | 17 | 3 | 0.0685 | 0.0850 | **0.0358** |
| *OSR2* | 8 | 1 | 0.2995 | **0.0196** | **0.0361** |
| *INHBA* | 7 | 3 | **0.0310** | 0.1945 | **0.0368** |
| *FOLH1* | 11 | 5 | 0.0695 | 0.0880 | **0.0373** |
| *FOXF2* | 6 | 3 | **0.0082** | 0.9510 | **0.0455** |
| *COL11A1* | 1 | 7 | **0.0117** | 0.6680 | **0.0459** |
| *KRT14* | 17 | 3 | **0.0098** | 0.8200 | **0.0468** |
| *FGF5* | 4 | 2 | 0.0865 | 0.0975 | **0.0487** |
| *SPAM1* | 7 | 1 | **0.0278** | 0.3775 | 0.0582 |
| *DLX2* | 2 | 2 | 0.9185 | **0.0120** | 0.0609 |
| *FOXE1* | 9 | 7 | 0.2570 | **0.0431** | 0.0610 |
| *HOGG1* | 3 | 3 | 0.5720 | **0.0212** | 0.0657 |
| *FGFR1* | 8 | 6 | **0.0339** | 0.3855 | 0.0697 |
| *SATB2* | 2 | 7 | 0.3850 | **0.0362** | 0.0735 |
| *PEX7* | 6 | 3 | **0.0334** | 0.4435 | 0.0772 |
| *PVRL1* | 11 | 6 | 0.5480 | **0.0306** | 0.0853 |
| *RUNX2* | 6 | 6 | **0.0218** | 0.7985 | 0.0879 |
| *JAG1* | 20 | 4 | **0.0332** | 0.5920 | 0.0969 |
| *SNX3* | 6 | 2 | 0.9565 | **0.0219** | 0.1019 |
| *FGF10* | 5 | 5 | 0.5600 | **0.0375** | 0.1020 |
| *MTRR* | 5 | 5 | **0.0385** | 0.5845 | 0.1078 |
| *AIP* | 11 | 3 | **0.0358** | 0.6735 | 0.1140 |
| *ATIC* | 2 | 4 | 0.9300 | **0.0382** | 0.1540 |
| *DLX6* | 7 | 1 | **0.0391** | 1.0000 | 0.1658 |

^a^ Gene ID from NCBI Entrez Gene. Genes associated in both samples are boldfaced.

^b^ P-values ≤ 0.05 are boldfaced (the Fisher-combined p-values have not been Bonferroni-corrected).

^c^ The top six genes are shown in **Figure 3B**.
