# Supplementary material for: Maternal Genes and Facial Clefts in Offspring: A Comprehensive Search for Genetic Associations in Two Population-Based Cleft Studies from Scandinavia
Source: PLoS One. 2010 Jul 9;5(7):e11493. doi: 10.1371/journal.pone.0011493 (PMC2901336; doi:10.1371/journal.pone.0011493)
Supplement: Table S4 — HAPLIN results for iCL/P. (0.08 MB DOC) [file pone.0011493.s004.doc]

**Table S4.** HAPLIN results for iCL/P

| Gene ID ^a^ | Chromosome | Number of SNPs/gene | NORWAY iCL/P p-value ^b^ | DENMARK iCL/P p-value ^b^ | Fisher-combined p-value ^b, c^ |
| --- | --- | --- | --- | --- | --- |
| *GABRB3* | 15 | 7 | **0.0039** | 0.1322 | **0.0044** |
| ***DVL1*** | 1 | 1 | **0.0242** | **0.0498** | **0.0093** |
| *FLNB* | 3 | 5 | **0.0191** | 0.0924 | **0.0130** |
| *GAD2* | 10 | 3 | 0.9744 | **0.0023** | **0.0160** |
| *FZD8* | 10 | 2 | 0.8605 | **0.0029** | **0.0172** |
| *ATIC* | 2 | 4 | 0.6088 | **0.0042** | **0.0179** |
| *CYP1B1* | 2 | 6 | 0.4047 | **0.0075** | **0.0206** |
| *EGFR* | 7 | 6 | **0.0118** | 0.3629 | **0.0276** |
| *RECQL4* | 8 | 3 | 0.8601 | **0.0068** | **0.0359** |
| *CTNNB1* | 3 | 4 | **0.0200** | 0.2937 | **0.0360** |
| *ALX4* | 11 | 2 | **0.0178** | 0.3494 | **0.0378** |
| *APE1* | 14 | 4 | 0.3735 | **0.0167** | **0.0379** |
| *SLC7A11* | 4 | 5 | **0.0096** | 0.6793 | **0.0392** |
| *FOXP2* | 7 | 5 | **0.0149** | 0.4445 | **0.0398** |
| *JAG2* | 14 | 4 | 0.2066 | **0.0364** | **0.0443** |
| *TCOF1* | 5 | 3 | **0.0076** | 0.9937 | **0.0446** |
| *CCDC6* | 10 | 1 | **0.0483** | 0.1602 | **0.0454** |
| *NOTCH3* | 19 | 3 | **0.0109** | 0.7907 | **0.0496** |
| *ALX3* | 1 | 4 | 0.5082 | **0.0177** | 0.0515 |
| *CHL1* | 3 | 5 | 0.9877 | **0.0096** | 0.0537 |
| *EGR3* | 8 | 4 | **0.0437** | 0.2260 | 0.0555 |
| *LEF1* | 4 | 5 | 0.6757 | **0.0160** | 0.0599 |
| *RARB* | 3 | 6 | 0.3163 | **0.0357** | 0.0620 |
| *COL2A1* | 12 | 5 | 0.8940 | **0.0150** | 0.0713 |
| *TCF1* | 12 | 4 | 0.4905 | **0.0294** | 0.0756 |
| *PON1* | 7 | 10 | 0.7819 | **0.0185** | 0.0757 |
| *SOX1* | 13 | 2 | 0.3962 | **0.0420** | 0.0849 |
| *FGFR2* | 10 | 3 | **0.0190** | 0.9098 | 0.0872 |
| *SOX9* | 17 | 5 | 0.5424 | **0.0328** | 0.0895 |
| *SKI* | 1 | 1 | **0.0458** | 0.4391 | 0.0987 |
| *PTCH1* | 9 | 2 | **0.0478** | 0.4383 | 0.1020 |
| *FOLH1* | 11 | 5 | **0.0436** | 0.5364 | 0.1112 |
| *GDF1* | 19 | 3 | **0.0289** | 0.8185 | 0.1123 |
| *RARA* | 17 | 2 | 0.9758 | **0.0316** | 0.1383 |
| *MDR1* | 7 | 3 | **0.0439** | 0.8765 | 0.1639 |
| *LHX8* | 1 | 4 | **0.0478** | 0.8883 | 0.1765 |

^a^ Gene ID from NCBI Entrez Gene. Genes associated in both samples are boldfaced.

^b^ P-values ≤ 0.05 are boldfaced (the Fisher-combined p-values have not been Bonferroni-corrected).

^c^ The top six genes are shown in **Figure 3C**.
