# Supplementary material for: Maternal Genes and Facial Clefts in Offspring: A Comprehensive Search for Genetic Associations in Two Population-Based Cleft Studies from Scandinavia
Source: PLoS One. 2010 Jul 9;5(7):e11493. doi: 10.1371/journal.pone.0011493 (PMC2901336; doi:10.1371/journal.pone.0011493)
Supplement: Table S5 — HAPLIN results for iCP. (0.10 MB DOC) [file pone.0011493.s005.doc]

**Table S5.** HAPLIN results for iCP

| Gene ID ^a^ | Chromosome | Number of SNPs/gene | NORWAY iCP p-value ^b^ | DENMARK iCP p-value ^b^ | Fisher-combined p-value ^b, c^ |
| --- | --- | --- | --- | --- | --- |
| *FLNB* | 3 | 5 | 0.0556 | **0.0140** | **0.0064** |
| *HIC1* | 17 | 2 | **0.0064** | 0.1300 | **0.0067** |
| *EPHB2* | 1 | 6 | 0.0582 | **0.0153** | **0.0072** |
| ***ZNF189*** | 9 | 5 | **0.0304** | **0.0414** | **0.0097** |
| *OSR2* | 8 | 1 | 0.2363 | **0.0092** | **0.0155** |
| *MMP14* | 14 | 4 | 0.0962 | **0.0290** | **0.0192** |
| *FOXE1* | 9 | 7 | 0.0653 | **0.0470** | **0.0208** |
| *LIMK1* | 7 | 3 | 0.1093 | **0.0390** | **0.0275** |
| *MTHFD2* | 2 | 2 | 0.1088 | **0.0402** | **0.0281** |
| *FOLH1* | 11 | 5 | 0.2340 | **0.0201** | **0.0299** |
| *COL11A2* | 6 | 6 | 0.0723 | 0.0674 | **0.0308** |
| *ARNT* | 1 | 4 | 0.3484 | **0.0142** | **0.0312** |
| *CYP3A7* | 7 | 2 | **0.0151** | 0.3371 | **0.0319** |
| *CDH1* | 16 | 4 | **0.0320** | 0.2134 | **0.0409** |
| *FGF5* | 4 | 2 | 0.0946 | 0.0737 | **0.0416** |
| *INHBA* | 7 | 3 | 0.0600 | 0.1169 | **0.0418** |
| *NQO1* | 16 | 3 | 0.1403 | 0.0527 | **0.0437** |
| *KRT14* | 17 | 3 | **0.0083** | 0.9128 | **0.0444** |
| *APOC2* | 19 | 2 | 0.1250 | 0.0622 | **0.0455** |
| *PRRX2* | 9 | 3 | **0.0090** | 0.8718 | **0.0458** |
| *RARB* | 3 | 6 | **0.0087** | 0.9430 | **0.0477** |
| *TGFBR2* | 3 | 5 | 0.3828 | **0.0215** | **0.0477** |
| *FOXH1* | 8 | 3 | 0.1113 | 0.0761 | **0.0489** |
| *SPAM1* | 7 | 1 | **0.0245** | 0.3500 | **0.0494** |
| *ALK6* | 4 | 6 | **0.0144** | 0.6488 | 0.0532 |
| *IRF9* | 14 | 3 | 0.2390 | **0.0413** | 0.0554 |
| *RAI1* | 17 | 2 | **0.0153** | 0.6459 | 0.0555 |
| *XRCC3* | 14 | 2 | 0.3679 | **0.0310** | 0.0624 |
| *PEX7* | 6 | 3 | **0.0320** | 0.3768 | 0.0653 |
| *RUNX2* | 6 | 6 | **0.0133** | 0.9660 | 0.0688 |
| *FGF12* | 3 | 6 | **0.0169** | 0.7624 | 0.0691 |
| *HIF1A* | 14 | 5 | **0.0418** | 0.3165 | 0.0704 |
| *DLX5* | 7 | 4 | **0.0187** | 0.8340 | 0.0803 |
| *FGF10* | 5 | 5 | 0.3933 | **0.0403** | 0.0815 |
| *GPR51* | 9 | 8 | **0.0246** | 0.6536 | 0.0824 |
| *TP63* | 3 | 9 | **0.0371** | 0.4734 | 0.0885 |
| *BMP10* | 2 | 3 | 0.8809 | **0.0204** | 0.0901 |
| *BMP2* | 20 | 4 | 0.9591 | **0.0200** | 0.0950 |
| *BCL3* | 19 | 2 | **0.0489** | 0.4129 | 0.0990 |
| *FOXF2* | 6 | 3 | **0.0213** | 0.9982 | 0.1031 |
| *DLX6* | 7 | 1 | **0.0486** | 0.4480 | 0.1050 |
| *CYP1A2* | 15 | 2 | 0.5522 | **0.0426** | 0.1118 |
| *ZFHX1B* | 2 | 5 | 0.5422 | **0.0491** | 0.1231 |
| *RFC1* | 4 | 3 | **0.0352** | 0.7559 | 0.1231 |
| *ESR1* | 6 | 8 | **0.0429** | 0.7858 | 0.1479 |
| *SNX3* | 6 | 2 | 0.9734 | **0.0432** | 0.1752 |
| *FGF1* | 5 | 4 | **0.0484** | 0.9669 | 0.1900 |

^a^ Gene ID from NCBI Entrez Gene. Genes associated in both samples are boldfaced.

^b^ P-values ≤ 0.05 are boldfaced (the Fisher-combined p-values have not been Bonferroni-corrected).

^c^ The top six genes are shown in **Figure 3D**.
